# The Doctor in Court

**Published:** 1898-10

**Authors:** George L. Reinhard

**Affiliations:** Ex-Judge Appellate Court of Indiana, Acting Dean of Department of Law, Indiana University, Bloomington


### Selections and Abstracts.

### THE DOCTOR IN COURT.

BY GEORGE L. REINHARD,

Ex-Judge Appellate Court of Indiana, Acting Dean of Department of Law, Indiana University, Bloomington.

There is a general impression abroad that somehow lawyers occupy a more favorable position in court than other professional men. Of course the lawyer in the practice of his profession occupies a peculiar official relation to the judge, which he does not occupy when he appears in court as a litigant or a witness. But in the latter relations he stands precisely on the same footing as the doctor or professional man whose occupation requires the exercise of special skill or knowledge. And so the liabilities and responsibilities growing out of the practice of medicine are much the same as those growing out of the practice of any other profession under similiar conditions and circumstances.

There are two ways, and only two, in which a professional man, whether he be a lawyer or a doctor, may be required to come into a court of justice, and they are, first as a party, and second as a witness. He may come into court voluntarily as a plaintiff to recover compensation for his services, or he may be brought in against his will, as a defendant. The doctor's appearance in the latter instances occurs most generally in cases of alleged malpractice.

Every physician and surgeon who attends a patient professionally sustains to such patient, or to his or her guardian, father, mother or husband, a contractual relation. It is in effect as if the responsible party had said, "Come and treat me for this disease," or "Go and attend my wife or child," or "Ward in this affliction or trouble. Do the best you can for him or her, and I will see that you are paid for your services whatever they are reasonably worth." The nature of the contract is precisely the same as that between an attorney and his client. In neither case does the professional man warrant or insure success, unless there be a special contract to that effect. No man is bound to undertake the performance of an impossibility. If a disease is incurable and the physician undertakes to cure it, he can not be held in damages for failure to cure, for that was beyond his skill to accomplish, even if he were among the formost of his profession. But if he guarantees a cure and does not perform it, he can recover no compensation, although it were impossible to perform such cure, for it was upon this condition alone that he contracted, and the law never undertakes to make a new or different contract for the parties from that they have voluntarily entered into. If the physician enter into such a contract he can not recover, even though he exercises the highest skill, ability and care in the treatment.

The lawyer may undertake to forclose a mortgage, or the surgeon to remove a limb, or the physician to treat a disease successfully, and if he thus warrants success, he can recover nothing if he fails, for it was his own fault that he assumed such an obligation. In the absence of such warranty, however, he will be allowed a reasonable compensation for his services. To this there is but one exception. If he fails to possess the necessary skill and to exercise the necessary care required in such circumstances, and injury results, he not only loses his compensation, but he renders himself liable to an action in damages for any injury sustained by reason of his ignorance or want of proper care.

Every professional man who holds himself out to the world as such, impliedly warrants that he has the required knowledge and skill, and will exercise the required care in the case in which he is employed. Every doctor who undertakes to treat a patient, impliedly contracts that he possesses the skill, ability and experience ordinarily possessed by those who practice the same profession. In the language of Justice Story: "In all these cases, where skill is required, it is to be understood that it means *ordinary* skill in the business or employment which the bailee undertakes, for he is not presumed to engage for extraordiary skill, which belongs to a few men only, in his business or employment, or for extraordinary endowments or acquirements. Reasonable skill constitutes the measure of the engagement in regard to the thing undertaken." And as Lord Chief Justice Tindall says: "Every person who enters into a learned profession undertakes to bring to the exercise of it, a reasonable, fair and competent degree of skill."

But it may be, and often is, a difficult matter to determine what is an "ordinary degree of skill." To determine this is to determine the standard of the profession. In some localities a much higher standard exists than in other localities.

In sparsely settled neighborhoods the physicians are scarce, and may have to be brought for a distance of many miles to the bedside of the sick. In such cases the patient impliedly engages that he will be satisfied with the best treatment which may be given him under the circumstances, and hence the same skill and experience would not be required as in a large city, where physicians are found in great numbers, and where medical schools and other facilities afford constant aid for improvement. It may be stated, therefore, with some degree of certainty, that a damage suit for malpractice is a more dangerous thing to the city physician than it is to the doctor of the small town or village or country settlement.

The standard of any profession anywhere is just what the members make it. The high-minded physician or lawyer, or other professional man—the man who has at heart the good of the community, as well as his own success, will always strive for a higher standard. Fortunately for your profession in this State, you have succeeded much better in securing the aid of the law in the elevation of its standard than we lawyers have in ours. Suppose there were such a thing as a constitutional provision that every person of good moral character, being a voter, shall be permitted to practice medicine, without any other qualifications, just as we have a constitutional provision that every person of this description shall be allowed to practice law. In that case a merciful Providence alone would be able to save the poor patients. But it is equally important that one should possess something more than a good moral character to practice the profession of law, where he may have in his keeping the lives and fortunes of those who come to him for advice and for services. But as no constitutional provision has stood in your way, you have steadily forged ahead, until now in Indiana the standard of the medical profession, I am ashamed to acknowledge it, has risen to a much higher plane than that of my own. Upon this achievement you are to be congratulated, and we can only hope that the time is not far distant when the Legislature shall have the right to pass a law requiring corresponding skill and experience in the practice of the legal profession.

But the physician and surgeon impliedly undertakes something even beyond the possession of ordinary skill, experience and ability. He also impliedly undertakes to use reasonable and ordinary care and diligence in applying the skill and ability of which he is possessed, for if he is ever so well informed, if he has had the very best of medical training, and the ripest of experience, yet if he is negligent and careless in the treatment of his patient, and injury results, he will be liable in damages. It will be noted that it is not *extraordinary* care and diligence that he undertakes to exercise, but only ordinary care and diligence, or, in other words, such care and such diligence as a reasonably prudent physician or surgeon would exercise in the same circumstances. The law will not hold a physician or surgeon to the exercise of the highest degree of care and skill, any more than it will hold the lawyer or other professional man to such exercise although morally they may both be bound thereto.

The doctor also undertakes, impliedly, to exercise his best judgment in the treatment of the disease. Whenever the physician or surgeon applies his best judgment in both diagnosis and treatment, it is all that can be reasonably required of him, it being of course presupposed that he possesses the skill and is exercising the degree of care and diligence already referred to. What is good judgment may, of course, not always be clear. But the physician and surgeon is not responsible for the errors of his judgment, provided the same is properly enlightened according to the facilities afforded the profession in his community.

Sometimes it is an error of judgment to amputate a limb, and the patient loses such limb as a result of such error. At other times the exercise of judgment may be erroneous in not performing an amputation when it should be performed. In either case, if it be a mere error of judgment and not due to rank ignorance or want of proper care, the physician or surgeon will be excused.

Another rule is that whatever is well and clearly settled must be known to the practitioner and must be applied by him.

Thus, if a patient's disease were such as knowingly required opiates in its treatment and the doctor were to prescribe *belladonna* and an injury should result, the physician would be liable. And so if amputation were clearly required and the surgeon failed to perform it he would be obliged to respond in damages.

But the patient also has a duty to perform. He must follow the directions of the doctor. In other words, he must "take his medicine," and if he fails to do so he can not hold the physician accountable. "Nothing can be more clear," says the Supreme Court of Pennsylvania, "than that it is the duty of the patient to co-operate with his professional adviser, and to conform to the necessary prescriptions; but if he will not, or under the pressure of circumstances he can not, his neglect is his own wrong or misfortune. No man may take advantage of his own wrong, or charge his misfortune to the account of another." But a surgeon can not delegate the duty of dressing or caring for wounds to incompetent nurses or attendants, and if he does so, it is at his own risk. "He must either dress the limb himself, or see that it is correctly done." The same is true of administering anesthetics in cases of surgical operations. If he leaves this task to an unskillful and careless attendant when he is the surgeon in charge, he will be responsible for the results.

The law also requires the physician or surgeon to devote as much time to the treatment of his patient as is reasonably necessary. It will not do for him to neglect one patient, in order to be at the bedside of another. Hence you may say that it is against the law for a doctor to have too many patients. Neither will he be allowed to go in pursuit of other callings and leave his patients unattended. He should devote his entire attention to the practice of his profession. He must not have too many irons in the fire. This is, of course, equally true of the attorney or other professional man, whose duties require him to remain at his post. He must fulfill his professional engagements at all hazards. And so it were better for the physician who has too much other business to attend to, so that he can not devote the greater part of his time to his profession, to abandon it entirely in order to rid himself of the consequences which will be likely to follow his negligences in case he undertakes to serve too many masters.

You will, of course, not expect me, and I certainly have not the capacity, in this short paper to give even an outline of what the physician or surgeon must do to escape the ordeal of a suit for malpractice. To do so would indeed be equivalent to writing a treatise upon the duties of the doctor to his patients, a task which is far more adapted to your profession than to mine. But it may not be out of place to state that in the application of legal principles to the science of medicine and surgery, the employment of a reasonable amount of common sense is a most exeellent criterion. If the professional man in the discharge of his duties as such will only act conscientiously and give his patient the benefit of all his skill, energy and reasonable care, he will rarely be liable to commit a fatal error, or one for which the law will hold him accountable in damages, provided always, that he has the necessary ability and training that fit him for a reasonably successful career in his profession.

But perhaps the more important business for which the medical man or surgeon will find himself invited into court, is not in his own but in some other person's lawsuit or case. Some other medical man or surgeon has a case of malpractice, or a claim for services, or some one is being prosecuted for a crime and the doctor is summoned into court as a professional witness, and unless he understands pretty well the underlying principles of medical jurisprudence, or knows something of the rules governing a court in the conduct of a trial, such as this, he will be laboring under great disadvantages, however eminent he may be in his profession.

"The whole subject of medical evidence," says Professor Elwell, "has been too much neglected by the medical profession at large. The witness stand should be the arena upon which the scientific man should gladly appear, as the public vindicator of justice; thereby defending and vindicating his own noble profession from the discredit brought upon it by the illiterate hangers-on who claim to represent it, but do not any more truly than does the miserable pettifogger truly represent the high-minded, intelligent and honorable lawyer."

But instead of this condition of things it is well known that the expert witness of late years is becoming more and more a tool for the lawyer or litigant to establish some pet theory in a case. Unfortunately it is too true that one can prove almost anything by an expert witness. For this purpose the witness is coached and drilled beforehand, so as to be able to meet the particular emergency for which he is wanted. If it is understood that "his side" desires to prove that a stagnant pool will produce malaria it is easy for him to furnish the evidence. If, on the other hand, it is desired to show that a pool of water, though stagnant, will absorb, and thus abate malaria, it is easy enough to find a witness who will prove it.

The genuine medical witness should never take into consideration the fact that "his side" would like for him to testify so and so. He should pass upon questions of a professional character and give his opinion much as a judge decides a case, free from bias, and indifferent as to whether the result benefits the one side or the other.

Dr. Coventry in a report to the American Medical Association, made many years ago, makes the following wise suggestions to the medical witness:

"*First.* He should listen attentively to the testimony, as to all the facts in the case, and avail himself of every authentic means of forming a correct opinion.

"*Second.* He should studiously guard against being biased, either by popular clamor or because he is called by one side rather than the other. He is to form his opinion exclusively from what appears in evidence, excluding, as far as possible, any previous prejudice, or what he may have seen in the papers or heard from rumor.

"*Third.* The medical witness is not to take into consideration the influence which his testimony may have on the prisoner at the bar, or the case under consideration, if he is testifying as to facts. If it is a matter of opinion, drawn from the facts, he should state it honestly; but if he has doubts, he should express them.

"*Fifth.* A medical witness should not assume the province of the jury, as to say a particular wound was the cause of death; he should only state what would be the ordinary effect of such a wound; or in a question of insanity, that the testimony given was an evidence, or was not an evidence, of insanity.

"*Sixth.* The medical witness should have his mind fully prepared before taking the stand as to what he can testify to, and his reasons, if they are required. He should, in his testimony, avoid, as much as possible, the use of technical or professional terms, which the jury would not be likely to understand; but, if unavoidable, then their meaning should be explained to the jury. In giving his testimony, he should keep cool and collected, and not permit himself to be irritated or confused by the counsel, and should avoid introducing any expression of opinion not immediately connected with the cause before the court."

I am of opinion that perhaps the last of these suggestions is the most important. Too much emphasis can not be placed upon the necessity of thorough preparation upon the subject in hand. The lawyers who conduct the examination on both sides will be apprised of the trend of the medical testimony in advance, and if they are men of standing in their profession, you may rest assured that they will be thoroughly informed. If you are not sure of your ground, the cross-examination will be a most trying ordeal to you, and it would have been far better for your reputation if you had not gone upon the witness stand.

Another thing which Dr. Coventry suggests can not be too often and too strongly recommended, and that is the avoiding of the too frequent use of technical terms. There is a tendency among all of us to exhibit a desire to display what we know professionally, but it always has a result opposite to what we expect it to have. It is just as easy to say the man was unconscious, as to say that he was *comatose*, and it will be much more easily understood. A witness who is plain and common-sensed in his conduct on the stand, who avoids all attempts at ostentation and display of learning, will always secure the respect of court and jury, and carry conviction to his hearers.

The necessity of keeping cool and collected, too, is of great importance to the medical witness, and ought to be kept constantly in mind by him. He should never allow himself to become confused or irritated by the questions of counsel. If any question is asked him which he is unable to answer, he should frankly say so, and not get himself more deeply into the dilemma by any attempts to explain what is not clear to him. If the question is obscure or ambiguous, he should ask the counsel to make it clear. He has a perfect right to this, and the court will always accord to every witness the rights which are due him. Even if counsel are guilty of impertinence or insolence to the witness, he can better afford to keep cool than to make a display of temper, for by the former course he always gains sympathy and approval, while by the latter he loses it. It is sometimes very difficult for expert witnesses to understand why certain questions are allowed to be asked of them by the court. It appears to them that such questions are a reflection upon their integrity and honesty. While lawyers doubtless frequently overstep the boundary of propriety in asking such questions, the court is not always at liberty to suppress them, as they may possibly lead to something throwing light upon the witness's capacity, fitness or candor, and if the court rules the question proper, it is the part of wisdom for him to answer, even though it involves to some extent an assumption of a fact which, if true, would be to the witness's discredit. The witness can well afford in such case to let the court and jury decide whether there is any occasion for the insinuation of the interrogating lawyer, and he will leave the stand with more credit than he would if he were to stop and quarrel with his questioner. Sometimes lawyers are desirous that the witness should become irritated, and should flounder, and if he does so, they have accomplished just what was desired. The professional witness will therefore often help his cause more by submitting to what seems to him is an indignity, than by refusing to answer or by answering evasively or curtly. Of course, no judicious judge will permit the witness to be badgered and harassed by foolish and impertinent questions. The witness should never lose sight of the fact, that it is the court and not the counsel, to whom he is responsible, and to whom he must look for guidance, and his entire conduct should be with this end in view. He can safely appeal to the court for protection if he needs it, but a too frequent exercise of this privilege will not be beneficial to him, if the court should happen to rule against him. As said by a standard writer: "The counsel who improperly invades the domain of a witness, either to embarrass or abuse him, will receive no sympathy from a court or jury, if the witness maintains a dignified manhood; but, on the other hand, such counsel will most certainly prejudice his client's cause, and impair his influence with the jury and court."—*The Medical and Surgical Monitor.*

